# Association of PKCζ Expression with Clinicopathological Characteristics of Breast Cancer

**DOI:** 10.1371/journal.pone.0090811

**Published:** 2014-03-06

**Authors:** Jian Yin, Zhipei Liu, Haixin Li, Jingyan Sun, Xinzhong Chang, Jing Liu, Shanshan He, Binghui Li

**Affiliations:** 1 Department of Breast Surgery, Tianjin Medical University Cancer Institute and Hospital, National Clinical Research Center of Cancer, Tianjin, China; 2 Key Laboratory of Cancer Prevention and Therapy, Tianjin, China; 3 Key Laboratory of Breast Cancer Prevention and Therapy, Tianjin Medical University, Ministry of Education, Tianjin, China; 4 Production and R&D Center, Tianjin Binhai Union Gene Technology Co. LTD, Tianjin, China; 5 Gene Bank, Union Stem Cell & Gene Engineering Co. LTD, Tianjin, China; 6 Tumor Tissue Banking Facility, Department of Epidemiology and Biostatistics, Tianjin Medical University Cancer Institute and Hospital, Tianjin, China; 7 Laboratory of Cancer Cell Biology, Tianjin Medical University Cancer Institute and Hospital, Tianjin, China; University of North Carolina School of Medicine, United States of America

## Abstract

The protein kinase C (PKC) family has been functionally linked to cancer. It has been suggested that atypical PKCs contribute to cell proliferation and cancer progression. With respect to breast cancer, PKCζ has been found to play a key role in intracellular transduction of mitogenic and apoptotic signals using mammary cell lines. However, little is known about its function *in vivo*. Here we examined the correlation between PKCζ protein levels and important clinicopathologic factors in breast cancer using patient samples. To conduct the study, 30 invasive ductal carcinoma cases and their paired normal tissues were used for tissue microarray analysis (TMA) and 16 were used for western blot analysis. In addition, the correlation between PKCζ expression levels and clinicopathologic characteristics was determined in 176 cases with relevant clinical data. Finally, the correlation between PKCζ and epithelial growth factor receptor 2 (HER2) expressions was determined using three breast cancer cell lines by western blot analysis. Both TMA and western blot results showed that PKCζ protein was highly expressed in primary tumors but not in paired normal tissue. The correlation study indicated that high PKCζ levels were associated with premenopausal patients (p = 0.019) and worse prognostic factors, such as advanced clinical stage, more lymph node involvement and larger tumor size. Both disease-free survival and overall survival rates were lower in the high PKCζ group than those in the low PKCζ group. No correlation was observed between PKCζ levels and age, histological grade, or estrogen or progesterone receptor expression status. A positive correlation between PKCζ and HER2 levels was observed in both tumor samples and cell lines. Our observations link PKCζ expression with factors pointing to worse prognosis, higher HER2 levels and a lower survival rate. This suggests that PKCζ protein levels may serve as a prognostic marker of breast cancer.

## Introduction

Protein kinase C (PKC) displays key regulatory roles in a wide variety of fundamental cellular processes, including signal transduction, regulation of gene expression and cell cycle control. Abnormal expression, activation and/or localization of PKC can dramatically alter cell growth status, inducing proliferation or apoptosis, which may cause various diseases including cancer [1]. A functional link between PKC and cancer is suggested by the fact that PKCs are major cellular receptors for the tumor-promoting phorbol esters [2], [3].

The PKC family is composed of 12 structurally related members, grouped into three subclasses: the classical (cPKC: α, βI, βII, γ), the novel (nPKC: δ, ε, η, θ) and the atypical (aPKC: ζ, λ/ι, µ). The last subclass is structurally and functionally distinct from the other PKC subclasses. They are not sensitive to diacylglycerol, calcium or phosphatidylserine, but are regulated by 3-phosphoinositides and phosphoinositide-dependent kinase 1 (PDK1) phosphorylation [4]–[6]. The unique N-terminal regulatory domain on aPKCs interacts with partitioning-defective (PAR)-3 and PAR-6 proteins, which play roles in asymmetrical cell division and cell polarization processes [7]. During cancer progression, this interaction is involved in the epithelial-to-mesenchymal transition that is characteristic of the invasive phenotype associated with metastatic carcinomas [8]–.

Breast cancer is the most common malignant disease in women globally. Previous studies, using immobilized mammary cell lines, have suggested that the aPKC isoform ζ (zeta) regulates intracellular transduction of mitogenic and apoptotic signals. However, little is known regarding the role of PKCζ in breast cancer *in vivo*. In this study, we investigated PKCζ expression in breast cancer tissue and analyzed the relationship of PKCζ expression levels with clinicopathological characteristics. Our data suggests that PKCζ may be used as a prognostic marker for breast cancer.

## Materials and Methods

### Patients and clinical specimens

All specimens used in this study were taken from a tumor tissue bank of the Tianjin Medical University Cancer Institute and Hospital. Invasive ducal carcinoma specimens used in this study were randomly selected from those obtained from female patients who had undergone mastectomy in 1996–1997 in the department of Breast Surgery at Tianjin Cancer Hospital. Survival status of patients was followed for up to 132 months post-surgery.

During the sample collection procedure of the tissue bank, samples were routinely cut into two parts for different treatments. Those for frozen were snap-frozen in liquid nitrogen, and then transferred and preserved in −80°C freezers. Those for fixation were rinsed in 10% formalin and then embedded in paraffin wax. Embedded samples were stored at room temperature. Specimens for protein isolation were snap-frozen and stored at −80°C, and those for immunohistochemistry (IHC) were fixed with 10% formalin and embedded in paraffin wax. Information on patient age, menopausal status, stage, tumor size at operation, lymph node status, histologic grade, estrogen receptor (ER), progesterone receptor (PR), and human epithelial growth factor receptor 2 (HER2) status, and relapse and survival times was retrieved from patient charts.

### Tissue microarray

The tissue microarray (TMA) was constructed from paraffin-embedded blocks of 30 paired invasive ducal carcinoma cases using a Beecher tissue arrayer (Beecher Instruments Inc., Sun Prairie, WI, USA). In brief, donor blocks were prepared after a thorough evaluation of hematoxylin and eosin-stained slides. One representative section of tumor or normal tissue for each cancer case was selected after the identification of representative tumor areas in each whole-mount slide. A single core (0.6 mm in diameter) was punched from the selected part of each donor block, using a specific orientation and placed into a pre-molded recipient paraffin wax block. Consecutive 4-μm-thick sections were cut from the recipient blocks and placed onto adhesive-coated slides for IHC analysis.

### Immunohistochemistry and image analysis

IHC was performed using VECTASTAIN® ABC Kit as described by the manufacturer (Vector, Burlingame, CA, USA). In brief, after dewaxing and rehydration, array sections and tissue slices were pretreated with EDTA and H_2_O_2_ for antigen retrieval and for quenching endogenous peroxidase activity. Then the sample slides were blocked with 2% bovine serum albumin (BSA), and incubated with a primary antibody against PKCζ (1∶50 at 4°C overnight; Santa Cruz Biotechnology, Dallas, TX, USA) and secondary antibody successively. Slides were visualized using 3, 3-diaminobenzidine (DAB) and lightly counterstained with hematoxylin. Negative controls were obtained by omitting the primary antibody.

Images were graded according to the percentage of tumor cells stained and the intensity of the staining. The percentage of cells stained was scored from 0 to 4+ by calculating the percent of positive to total epithelial cells in an area covering 25% of the tumor. In brief, 0 for 0% positive, 1+ for 25% and less, 2+ for more than 25% but less than or equal to 50%, 3+ for more than 50% but less than or equal to 75% and 4+ for more than 75%. The intensity of immunostaining was scored semiquantitatively as: 0 for no obvious yellow particles in epithelial cell plasma membrane or cytoplasm; 1 for weak (light yellow particles); 2 for moderate (moderately yellow particles); 3+ for strong (deep yellow particles). Two pathologists independently determined scores for all samples. In cases where the difference in score exceeded two, the slides were re-examined until the pathologists reached an agreement.

The scores of percentage of positive cells and intensity of staining from the same specimen were added. When the sum was greater than or equal to 3, the staining of the specimen was taken as positive.

### Protein isolation and western blot

Protein was isolated from cell lines or tumor and their paired normal tissue samples using lysis buffer containing 1% SDS, 10 mmol/L Tris-Cl (pH 7.6), 150 mmol/L NaCl, 20 g/L aprotinin, 20 g/L leupeptin, and 1 mmol/L phenylmethanesulfonyl fluoride. Protein lysates (30 μg/lane) were separated on 10% SDS-PAGE under denatured conditions, and transferred to PVDF membrane. Membranes were blocked with 1% BSA. Immobilized proteins were probed using a primary antibody against PKCζ (sc-216, Santa Cruz Biotechnology), followed by an HRP conjugated secondary antibody. Immunoreactive proteins were visualized with an enhanced chemiluminescence detection system (Amersham-Pharmacia Biotech, Piscataway, NJ, USA). The optical densities of western blot bands were analyzed with Image J software and statistically analyzed by t-test.

### Statistics

Statistical analysis was performed using the Statistical Package for the Social Sciences software (SPSS, Chicago, IL, USA). The Chi-square test and Fisher's exact test were used to examine the association between PKCζ expression and various clinicopathological parameters.

### Ethical statements

All experiments and information collected were approved by the research ethics committee of Tianjin Medical University. All specimens were taken from the tumor tissue bank of the Tianjin Medical University Cancer Institute and Hospital. Data were analyzed anonymously.

## Results

### PKCζ was expressed highly in human breast carcinomas

**Figure 1:**
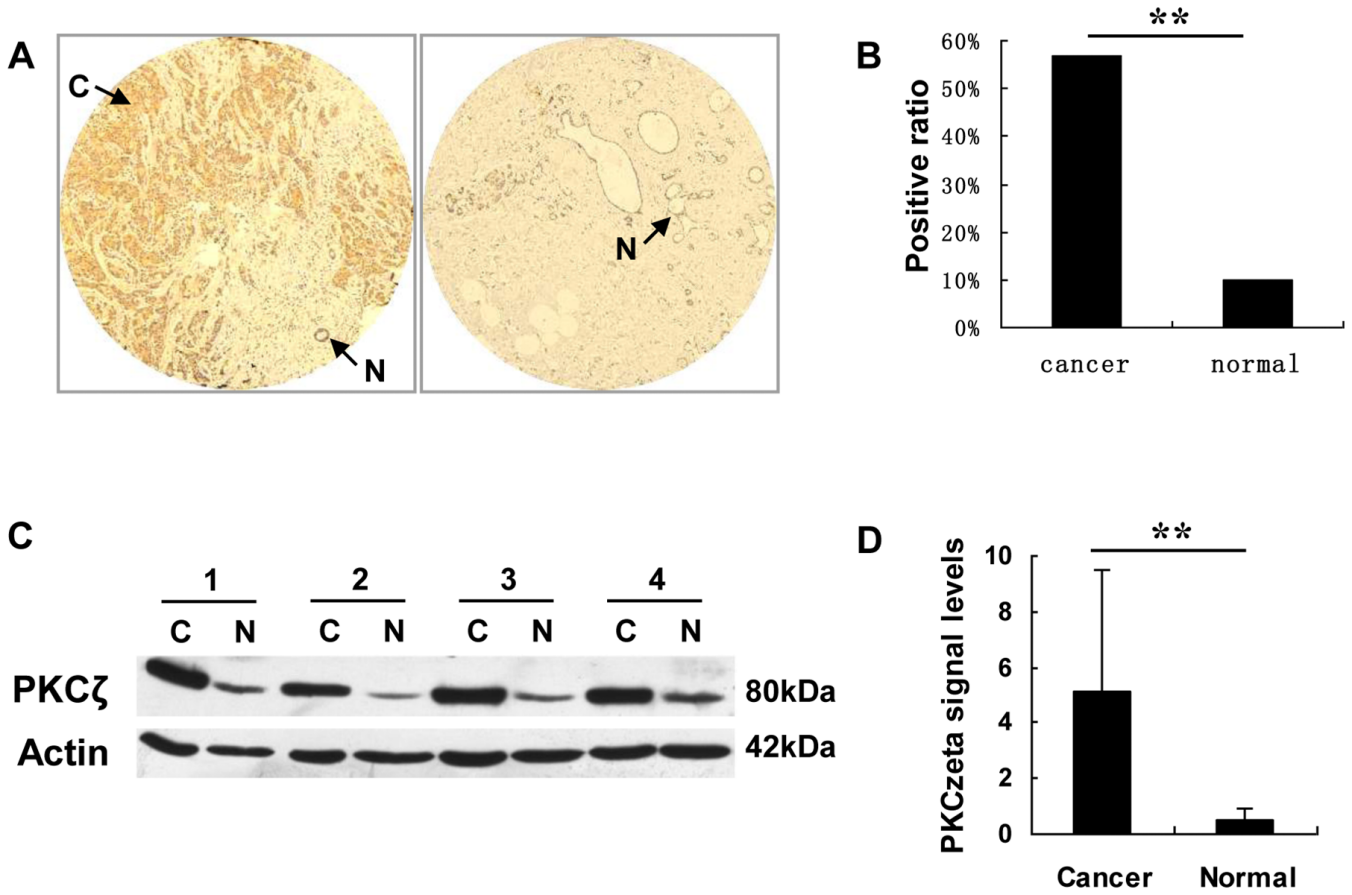
PKCζ is expressed strongly in invasive tissue, but weakly/not in normal tissue. A) IHC images of TMA analysis from a paired invasive ductal carcinoma case. Arrows point to areas of carcinoma (C) or normal (N) tissue. B) Positive ratio of PKCζ staining in TMA, 56.7% (17 of 30 cases) in invasive samples and 10% (3 of 30 cases) in normal samples. **, p<0.001 by Fisher's exact test. C) Western blots of PKCζ and actin from four paired cases; carcinoma (C) and normal (N) samples. D) Relative PKCζ levels in western blots. PKCζ levels were normalized by ß-actin levels as loading control. PKCζ levels were much higher in cancer than in normal specimens (5.09±4.42 vs 0.52±0.39). **, p<0.01 by t-test.

To investigate PKCζ expression in breast cancer, TMA and western blots were performed using paired samples from patients with invasive ductal carcinoma (Fig. 1). Compared with normal tissue, carcinoma tissue displayed much higher signals for PKCζ by both methods. Thirty paired samples were analyzed using TMA, and the number of invasive samples that scored positive for PKCζ (17/30, 56.7%) was much higher than that for normal samples (3/30, 10%) (p<0.01). Sixteen paired samples were analyzed by western blot. Only one single band around 80 kDa was detected by the antibody against PKCζ. Similarly, relative PKCζ levels (normalized by ß-actin) were much higher than those in normal smples (5.09±4.42 vs 0.52±0.39, p<0.01 by t-test).

### Relationship between PKCζ expression levels and pathological characteristics of breast carcinomas

**Figure 2:**
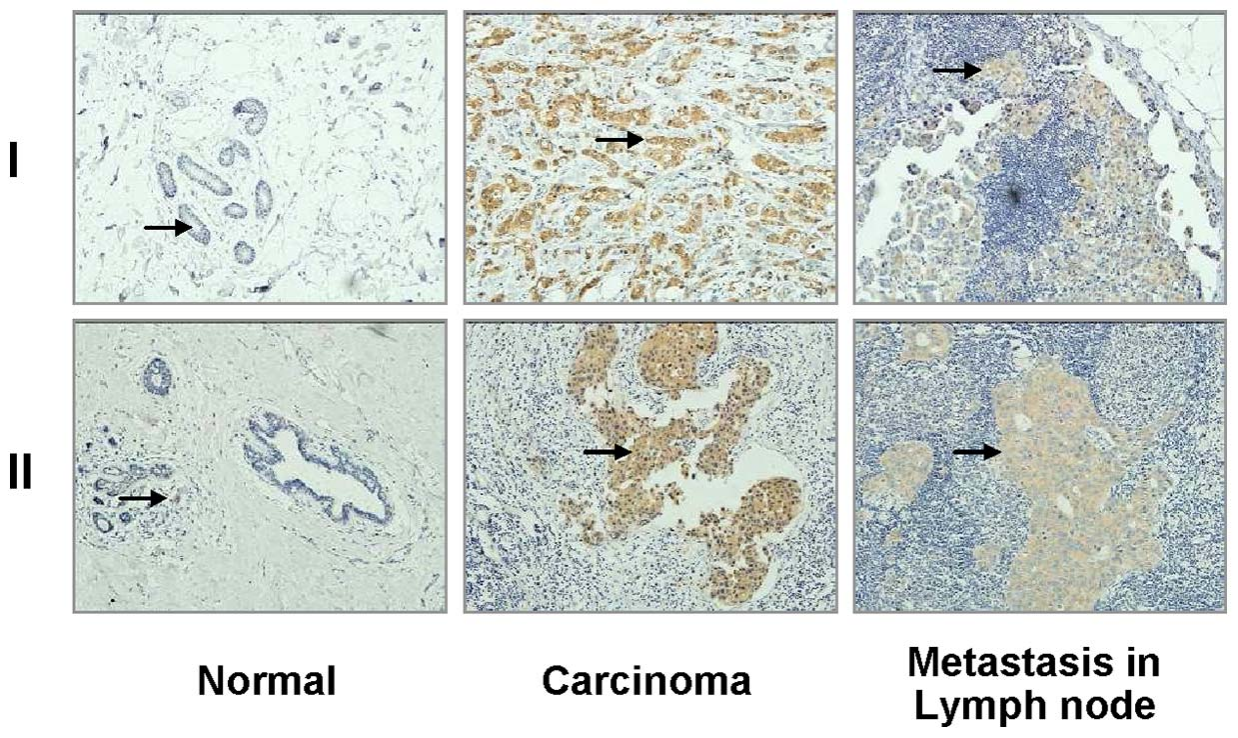
PKCζ expressed strongly in carcinoma tissue, but weakly/not in normal tissue and in lymph node metastasis. Samples of IHC images were from two independent individuals (I and II). Arrows point to PKCζ signals.

**Figure 3:**
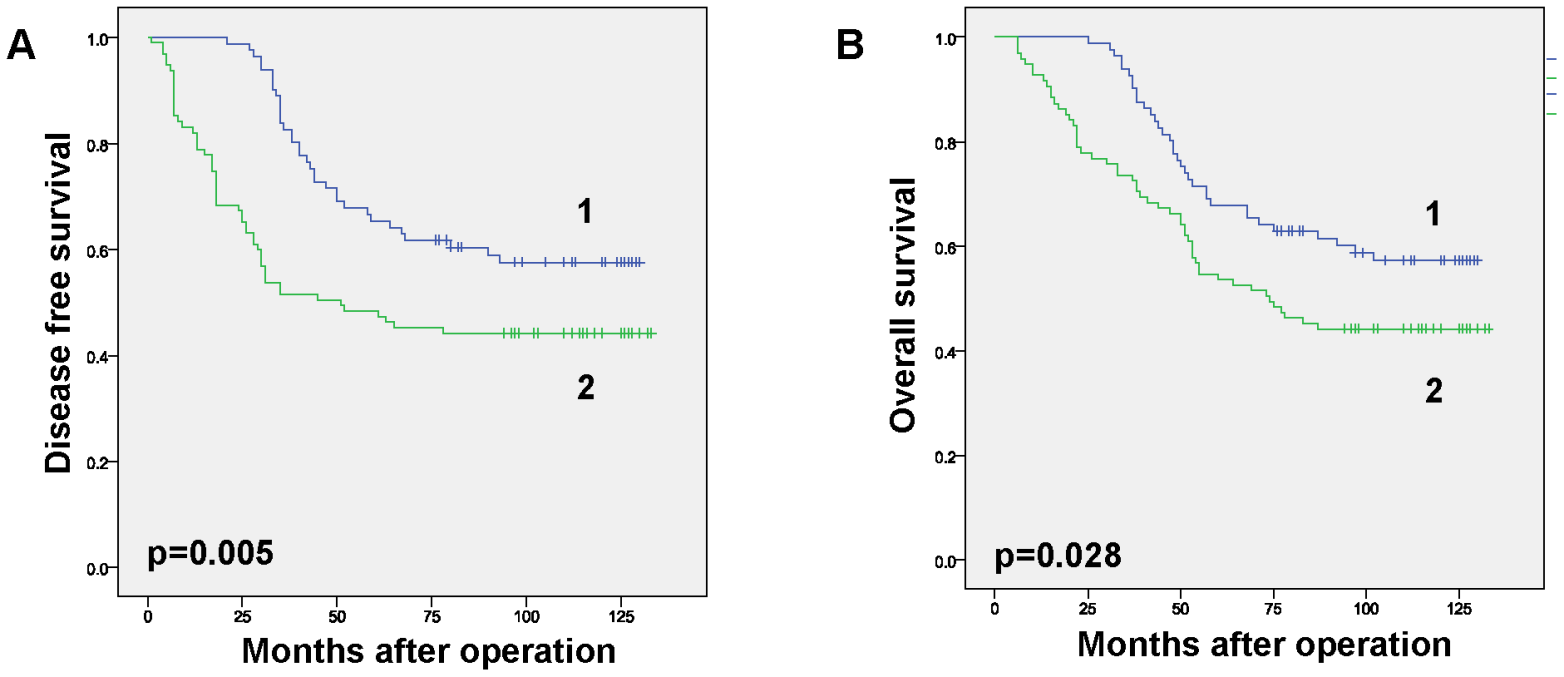
Survival analysis of breast cancer patients with different PKCζ levels. Survival status of patients was tracked for 132 months post-operatively. 1, low expression group; 2, high expression group. The survival statistical analysis was concluded by SPSS method. Survival curves were obtained using the Kaplan-Meier method. Differences in survival curves were assessed according to the log rank test. Differences were considered significant at values of P<0.05. **A)** Disease-free survival plot. **B)** Overall survival plot. Survival rates were calculated and obtained using SPSS software.

To analyze the relationship between PKCζ expression levels and pathological characteristics of breast cancer, samples from 176 patients with invasive ductal carcinoma were investigated. PKCζ levels were determined in tumor samples using IHC (Fig. 2). Patients were divided into two groups by PKCζ level, and statistically analyzed according to each clinicopathological parameter (Table 1). Between the low and high PKCζ expression groups, distribution of age, histological grade of tumor, and expression status of ER and PR showed no differences (p>0.05). Based on menopausal status, up to 62% of patients in the high PKCζ group were premenopausal, while 44.4% were in the low PKCζ group (p = 0.019). Of note, high PKCζ levels displayed significant correlation with factors indicating worse prognosis. Compared with the patients in the low PKCζ group, those in the high PKCζ group were in advanced clinical stages (55.7 vs 22.2% in stage III/IV, p<0.001), with more lymph node metastases (71.6 vs 46.1% positive, p = 0.0009) and larger tumor size (40.9±20.3 mm vs 27.2±10.7 mm, p<0.001). The correlation was further confirmed in the survival analysis. Both disease-free and overall survival rates were lower in the high PKCζ expression group (Fig. 3).

**Table 1:** Correlation between PKCζ expression and clinicopathologic characteristics of human breast carcinomas.

Clinicopathological parameters	PKCζ expression			t or x^2^	P value
	Low (+/−)	High (++/+++)			
	81	95			
**Age (y)**	52.3±9.7		50.9±10.2	0.739	0.814
**Menopause**					
** before**	36 (44.4%)		59 (62.1%)	5.490	0.019
** after**	45 (55.6%)		36 (37.9%)		
**Clinical stage**					
**I**	20 (24.7%)		8 (8.4%)	23.112	<0.001
**II**	43 (53.1%)		34 (35.8%)		
**III**	14 (17.3%)		46 (48.4%)		
**IV**	4 (4.9%)		7 (7.4%)		
**Lymph node metastasis**					
** Negative**	70 (86.4%)		39 (41.1%)	38.1683	<0.0001
** Positive ≥5**	11 (13.6%)		56 (58.9%)		
**Histologic grade**					
**1**	8 (9.9%)		3 (3.2%)	5.344	0.691
**2**	62 (76.5%)		70 (73.7%)		
**3**	11 (13.6%)		22 (23.2%)		
**Tumor size**	27.2±10.7		40.9±20.3	−4.612	<0.001
**ER**					
**Negative**	43 (53.1%)		57 (60.0%)	0.852	0.356
**Positive**	38 (46.9%)		38 (40.0%)		
**PR**					
**Negative**	47 (58.0%)		53 (55.8%)	0.089	0.765
** Positive**	34 (42.0%)		42 (44.2%)		
**HER2**					
** Negative**	60 (74.1%)		60 (63.2%)	12.784@	0.004
** 1+**	6 (7.4%)		1 (1.1%)		
** 2+**	9 (11.1%)		11 (11.6%)		
** 3+**	6 (7.4%)		23 (24.2%)		

@:Fisher's exact test.

### Positive correlation between PKCζ and HER2 levels

**Figure 4:**
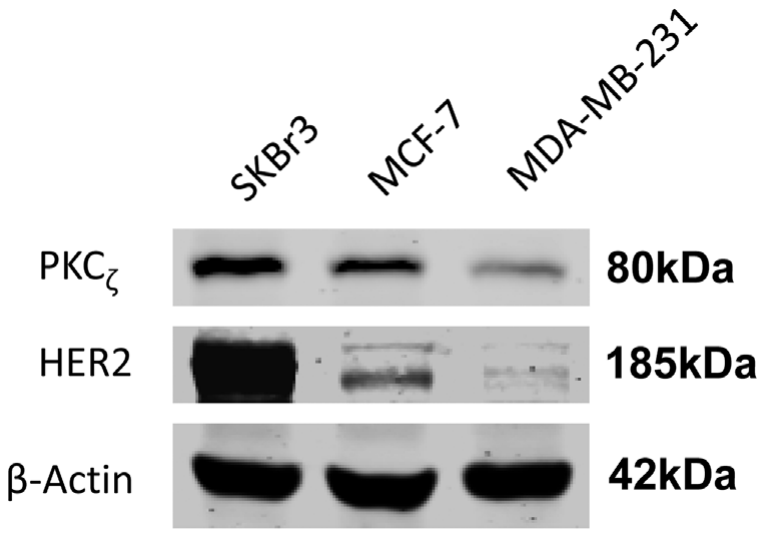
Positive correlation between PKCζ and HER2 levels in breast cancer cell lines. Expression of PKCζ and HER2 were determined using western blots. ß-actin served as a loading control.

Interestingly, our data suggested that high PKCζ levels were related to high HER2 levels. More patients in the high PKCζ group were HER2 positive (36.8 vs 25.9%, p = 0.004) (Table 1). The difference was further enlarged (24.2 vs 7.4%) when compared with the HER2 (3+) subgroup. To confirm our observation, we investigated PKCζ levels and HER2 levels in three breast cancer cell lines by western blot (Fig. 4.). The cell line data also indicated a positive correlation between PKCζ and HER2.

## Discussion

During our investigation, greater than 50% of breast cancer specimens overexpressed PKCζ, and PKCζ that were related to pathological and prognostic characteristics, including clinical stage, lymph node metastasis status, tumor size, HER2 status and survival rate. Our results were different from those studies in which transcript levels were determined. For example, Awadelkarim *et al.* found that PKCζ was overexpressed in only 9.6% of tumor specimens by qRT-PCR. Neither breast cancer molecular subtype, according to hormone receptor and HER2 status, nor relapse-free survival (RFS) was found to be related to PKCζ transcript levels [11]. One of the possible reasons might be that the antibody against PKCζ theoretically detected signals from other PKC forms, PKCλ and PKCι. However, molecular weights of those PKC isoforms were different. As it was indicated in the datasheets from manufacturer, PKCζ signals were around 80kDa, while signals for PKCλ and PKCι were around 74 kDa and 65 kDa, respectively. The specificity of the antibody against PKCζ has been conformed by several studies [12], [13]. Since we detected and analyzed only one single band around 80kDa, the merge signals from other PKC isoforms were unlikely the major cause. Another reason could be due to differences between PKCζ mRNA and protein levels, whereby elevated proteins levels may result from regulation of PKCζ expression downstream of transcription, such as protein translation, stability and solubility. Drosophila deficient in INAD (inactivation no afterpotential D) protein, which is required for the stability of PKC, caused a reduction in PKC protein [14]. Fas-associated protein with death domain (FADD) has been found to regulate cPKC phosphorylation and the subsequent change in stability and solubility of cPKC in human and mouse cells [15]. It has been reported that modification of proteins by phosphorylation, acetylation and redox reactions, altered not only the activity but also the stability of some proteins. The tumor suppressor p53 is a well-known example [16]. Further investigation is required to characterize PKCζ protein status *in vivo*.

Prognosis of breast cancer is affected critically by metastasis, in which cell motility and chemotaxis are involved [17]. Early studies linked PKCζ to chemokine-triggered cell migration, not only in multiple types of cancer cells [18]–[20], but also in metastasis-related macrophages [21]. Investigations with specific inhibitors against PKCζ also suggested that PKCζ is associated with cancer with respect to both chemotaxis and immune responses [22], [23]. Therefore, PKCζ may serve as a potential target for cancer therapy and as a prognostic biomarker. To date, several groups have shown that PKCζ regulates proliferation, invasion and metastasis of breast cancer cells *in vitro* [19], [24], [25]. There is also some evidence linking PKCζ with prognosis of several tumor types in patients, including non-gastrointestinal stromal tumor soft tissue sarcomas (non-GIST STSs) [26], renal cell carcinoma [27] and ovarian cancer [28]. Though overexpression of PKCζ has been observed in breast tumor tissue rather than in adjacent breast normal tissue, no consistent conclusion has been reported in breast cancer yet [29]. Our observation linked overexpression of PKCζ with advanced clinical stages, greater lymph node metastasis and increased HER2 expression, suggesting that PKCζ protein levels may be related to prognosis of breast cancer in patients. Since PKCζ has been reported to be a mitogenic downstream mediator of EGFR in cell signal transduction [30], inhibiting PKCζ could target HER2 or EGFR and potentially enhance breast cancer chemotherapy.
